# Detection of Salt Marsh Vegetation Stress and Recovery after the Deepwater Horizon Oil Spill in Barataria Bay, Gulf of Mexico Using AVIRIS Data

**DOI:** 10.1371/journal.pone.0078989

**Published:** 2013-11-05

**Authors:** Shruti Khanna, Maria J. Santos, Susan L. Ustin, Alexander Koltunov, Raymond F. Kokaly, Dar A. Roberts

**Affiliations:** 1 Center for Spatial Technology and Remote Sensing (CSTARS), Department of Land, Air and Water Resources, University of California Davis, Davis, California, United States of America; 2 Center for Spatial and Textual Analysis (CESTA), Stanford University, Stanford, California, United States of America; 3 United States Geological Survey, Denver, Colorado, United States of America; 4 Department of Geography, University of California Santa Barbara, Santa Barbara, California, United States of America; Albert Einstein College of Medicine, United States of America

## Abstract

The British Petroleum Deepwater Horizon Oil Spill in the Gulf of Mexico was the biggest oil spill in US history. To assess the impact of the oil spill on the saltmarsh plant community, we examined Advanced Visible Infrared Imaging Spectrometer (AVIRIS) data flown over Barataria Bay, Louisiana in September 2010 and August 2011. Oil contamination was mapped using oil absorption features in pixel spectra and used to examine impact of oil along the oiled shorelines. Results showed that vegetation stress was restricted to the tidal zone extending 14 m inland from the shoreline in September 2010. Four indexes of plant stress and three indexes of canopy water content all consistently showed that stress was highest in pixels next to the shoreline and decreased with increasing distance from the shoreline. Index values along the oiled shoreline were significantly lower than those along the oil-free shoreline. Regression of index values with respect to distance from oil showed that in 2011, index values were no longer correlated with proximity to oil suggesting that the marsh was on its way to recovery. Change detection between the two dates showed that areas denuded of vegetation after the oil impact experienced varying degrees of re-vegetation in the following year. This recovery was poorest in the first three pixels adjacent to the shoreline. This study illustrates the usefulness of high spatial resolution airborne imaging spectroscopy to map actual locations where oil from the spill reached the shore and then to assess its impacts on the plant community. We demonstrate that post-oiling trends in terms of plant health and mortality could be detected and monitored, including recovery of these saltmarsh meadows one year after the oil spill.

## Introduction

Ecosystems located in regions where oil and gas development occurs are highly susceptible to direct and indirect impacts of petroleum extraction and refining [1]. Coastal ecosystems have been disproportionately affected, because a majority of extraction sites are in coastal areas [2], as are the locations for ship transport and refineries. The Mississippi Delta contains the largest area of coastal wetlands in the United States but also supports one of the most extensive petroleum extraction operations in the world, exposing these wetlands to the impacts of oil contamination since the early 1900s [1]. The British Petroleum Deepwater Horizon (BP-DWH) Oil Spill in the Gulf of Mexico is the biggest coastal oil spill in US history and one of the five largest spills in the world by volume [3]. The impact of the BP-DWH spill on Gulf ecosystems is still under active examination [4], [5], but a long history of small and large oil spills in these regions has resulted in numerous studies on both impacts and recovery [6], [7], [8], [9], [10].

The salt marshes of Louisiana lost about 6500 hectares of wetland area per year between 1985 to 1989 due to subsidence, erosion, scouring, and other reasons [7]. Oil contamination can increase erosion and salt marsh loss due to the oil-induced plant mortality. The longer the residence time of the oil in the wetland, the greater is the impact of oil and slower is the recovery [2], [11], [12]. All weights of crude oil are highly toxic to plants through direct impacts on plant metabolism and indirectly through disruption of plant-water relationships, and reduced oxygen exchange between atmosphere and soil [1], [7], [8], [13]. Oil coating the leaves can obstruct or prevent gas exchange [1], [8], [14]. When leaves are oiled, mortality is much higher, especially in *Spartina* species, which are otherwise relatively resistant to oil impacts [1], [7]. Oil also affects the microbial community and nutrient cycling in the soil, which can have a negative impact on plant health [8].

**Figure 1:**
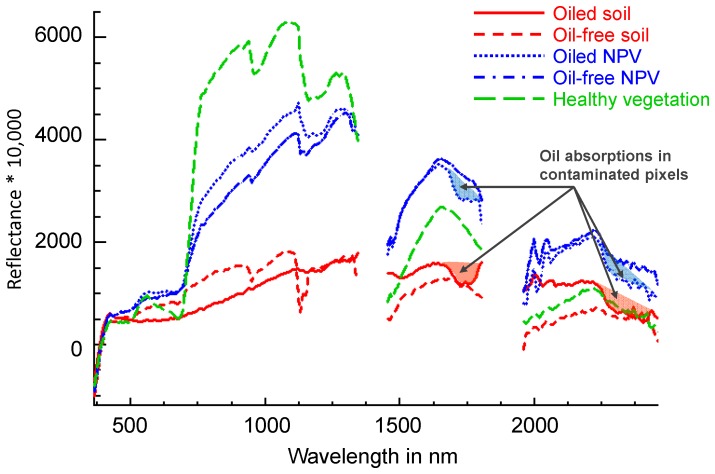
AVIRIS spectra from oiled and oil-free pixels. AVIRIS spectra for oiled and oil-free soil, oiled and oil-free non-photosynthetic vegetation (NPV), and healthy photosynthetic vegetation. Oil absorptions (centered at 1720 nm and 2300 nm) are visible in spectral signatures of oiled pixels. Bad band values are not displayed.

Remote sensing has proven to be a valuable tool for detection and mapping of marine oil spills [15], [16], [17], [18], [19], [20], [21], [22]. In terrestrial environments, reliable detection typically requires highly detailed spectral information provided by imaging spectrometers [18], [19]. While the presence of oil reduces soil reflectance in the visible part of the electromagnetic spectrum [23], other factors can lead to the same effect, for example, high organic matter content in soil [24]. The oil signal is characterized by absorption features in the near-infrared (NIR) and shortwave-infrared (SWIR) regions, due to overtones and combinations of C-H and C-O vibrational absorptions [20], [23], [25], [26], [27], [28] (Figure 1). In particular, the NIR 1730 nm and SWIR 2300 nm bands are best suited for detecting oil on bare soil as they do not overlap with absorption features of soil background materials [5], [23], [27], [29]. The 2300 nm absorption feature can be confused with carbonate absorption in soil [30], [31], but high salinity soils found in saltmarshes are acidic with very low carbonate content [32]. Both absorption features of oil overlap with cellulose-lignin absorption features found in the spectra of dead or senescent plants, often termed non-photosynthetic vegetation (NPV). However, the 2300 nm absorption has a unique spectral shape compared to NPV spectral features [5], [33]. As plants senesce and lose their leaves, the soil fraction in the pixel spectrum increases, and oil under the canopy can be detected.

The physiological changes due to plant stress can be measured by specific changes in the reflectance spectrum. As plants become increasingly stressed, they lose pigments, water, and ultimately leaves, all of which can be measured through changes to their spectral properties [34], [35], [36],[37],[38]. Plant stress can be detected by several different broadband and narrowband vegetation indexes that track plant pigment concentration [34], [37], water content [8], [39], [40], [41] and plant cover [38], [42], [43]. The spectrum of healthy photosynthetic vegetation has a characteristic “red edge” where the reflectance rises sharply between 680 nm (red) and 780 nm (near infrared) wavelengths. The red edge is the long wavelength edge of the chlorophyll absorption. The properties of the red edge change rapidly as the plant becomes more stressed [44], [45], [46], [47], [48], [49], [50].

Multiple studies since the BP-DWH spill have mapped the oil spill on the ocean surface [51], [52], [53], [54], [55] but very few studies have looked at the impact of this spill on wetland vegetation [4]. Mishra et al. [4] assessed the impact of the BP-DWH spill in East Bird's Foot, Louisiana using field spectra and Landsat imagery. However, the broad spatial and spectral resolution of multi-spectral data prevented the assessment of changes in the narrow absorption features necessary for direct oil detection. Hence the study did not examine the spatial proximity of vegetation stress to oil contamination. Large areas of vegetation “change” mapped with 30 m pixels could be due to other factors, e.g., phenological differences between years. The spectral resolution of spectroscopy data acquired at high spatial resolution is necessary to confirm the presence of oil and to attribute whether observed stress is due to oil or other stress factors [5], [44]. The Airborne Visible Infrared Imaging Spectrometer (AVIRIS) measures the full spectrum between 380 to 2500 nm in 224 sequential bands, each approximately 10 nm wide. It has been successfully used to map both oil contamination and vegetation stress [5], [44], [56].

The objective of this study is to determine the impact of the BP-DWH Oil Spill on the tidal salt marshes in Barataria Bay, LA, one of the areas heavily impacted by oil reaching the shoreline. We were interested in assessing: 1) the magnitude of any oil-induced stress on inland vegetation and 2) evidence for recovery or continued decline of contaminated vegetation communities the following year. We analyzed AVIRIS imagery flown over saltmarshes to determine the extent and the level of plant stress in proximity to oil contamination. Band indexes were used to determine the effect of oil on plant stress, as measured by changes in plant water and chlorophyll content. Finally, we used change detection between images collected approximately a month after the oil spill made landfall (September 2010) and one year later (August 2011) to examine whether marsh vegetation continued to decline or recovered from the immediate impact of the oil spill.

## Materials and Methods

### Field Data Collection

Field work was conducted by the United States Geological Survey (USGS) in Barataria Bay on July 10, 2010 and again on August 12–13, 2010 [5]. At each 2×2 m survey point, vegetation species composition, canopy condition, presence of oil, and penetration of oil into the marsh were noted. Locations of survey points were selected based on access to the marsh through gaps in protective booms. During the August survey, oil detected in a previous image acquired on July 31, 2010 was used to select areas of extensive oiling [5], [57]. Forty of the survey points were located in the Bay Jimmy region, which was analyzed for this study.

### Data Preprocessing

**Figure 2:**
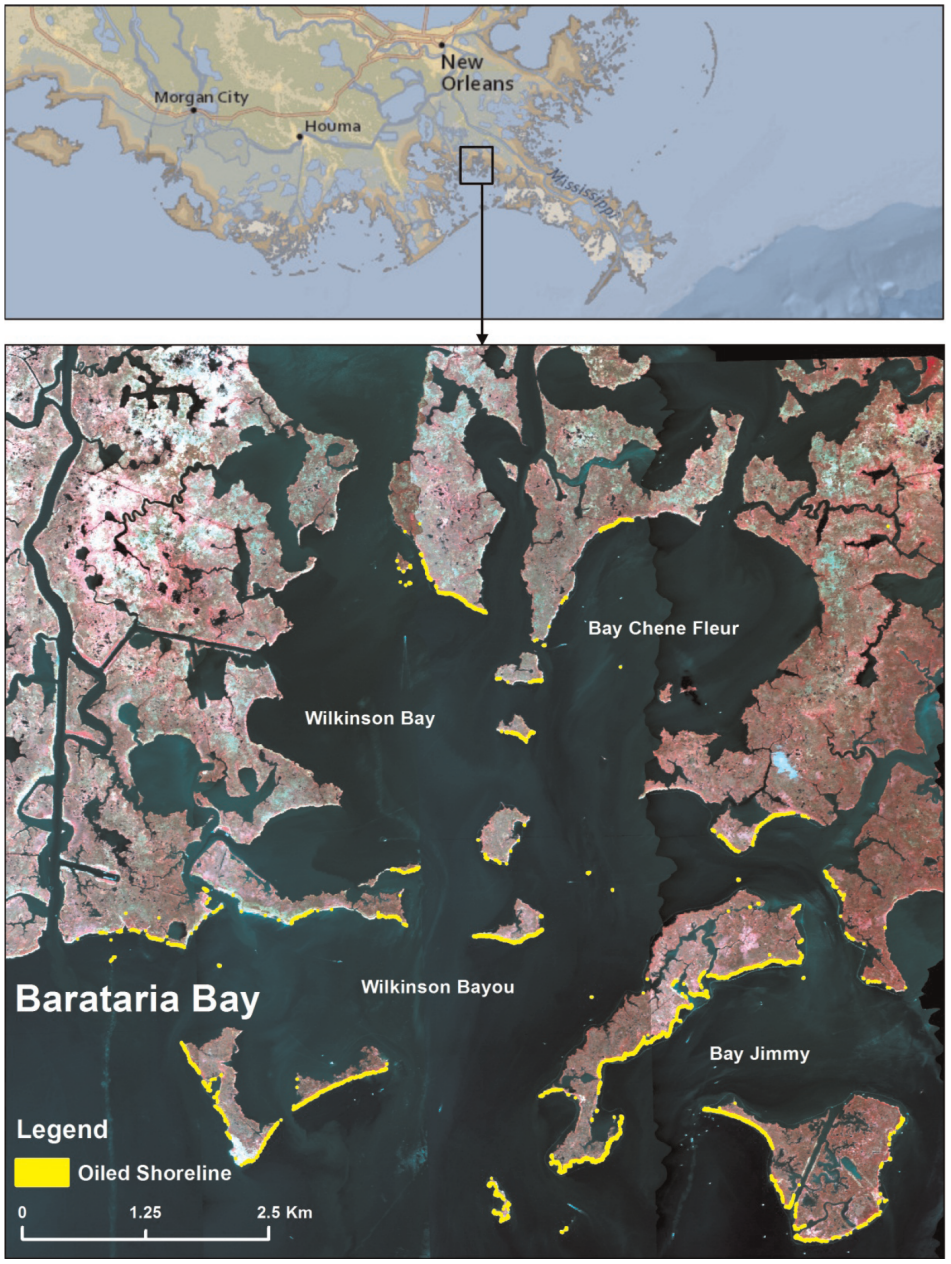
Color-infrared view of study site showing oiled shoreline. Location of Barataria Bay in the Mississippi Delta in the Gulf of Mexico, AVIRIS images of Barataria Bay depicted in color infrared bands with oiled shoreline shown in yellow.

AVIRIS image data were collected over the oil impacted regions of the Gulf of Mexico coastal wetlands from May to October of 2010 and from May to December of 2011. Flightlines that had all navigation and georectification information available and were at least 70% cloud free were selected for further processing. We focused on Barataria Bay (area 86 km^2^; Figure 2) because it was severely impacted by the oil spill. Data collected on two dates at different altitudes were chosen to study the progression of wetland impact and recovery: 1) September 14, 2010 (pixel resolution: 3.5 m), and 2) August 15, 2011 (pixel resolution: 7.7 m). We selected the September 2010 imagery because most of the oil had already come ashore and we hypothesized that the time delay between the arrival of oil and the response of the plant community would have passed. The August 2011 imagery was selected because it was closest in seasonality to the 2010 imagery. We analyzed four flightlines in September 2010 and two flightlines in August 2011 that covered the most heavily impacted region of Barataria Bay (Figure 2). The flightlines were atmospherically corrected using ACORN 6, mode 1.5 (ImSpec LLC, Seattle) to apparent surface reflectance. The September 2010 images were georectified to 1 m National Agricultural Imagery Program (NAIP) color infrared images collected in 2010. Even though we were planning to compare the September 2010 with the August 2011 imagery, we did not radiometrically calibrate the two images together, because our comparisons were based on index values. Indexes are relatively insensitive to differences in absolute reflectance since they compare relative differences between band values and are frequently normalized.

The AVIRIS image data used in this study are publicly available from the Jet Propulsion Laboratory (JPL) archive. Field data collected by USGS will be made available upon request.

#### Image co-registration

NASA Jet Propulsion Laboratory (JPL) provides AVIRIS images with georeferencing information derived from inertial navigation data and GPS. Nonetheless, images georeferenced based on this information often suffer from residual misalignment by a few pixels or more. With 3.5 and 7.7 m pixels of AVIRIS imagery used in our study, this misalignment translated into displacements of the order of dozens of meters, whereas surface (including vegetation) conditions near the shore were known to exhibit sharp gradients on the scale of just a few meters. Therefore, we co-registered the AVIRIS images using an automated image registration technique [58] that combines robust band-wise compensation for radiometric differences in images [59] with an iterative gradient-based video-sequence alignment method by Irani [60], under the affine image motion model. The method does *not* change the band values of either image. We resampled images from both dates to 1.1m and subset the two 2011 flightlines to the four 2010 flightlines (half the swath width because pixel size is smaller). Then all four sets of images were coregistered. As a result of the image co-registration, the residual pixel misregistration was markedly reduced, allowing more accurate analysis of the changes in vegetation conditions. The base images were selected to be the September 2010 images and the August 2011 images were co-registered to the base images. Areas of large or systematic change in the scene (e.g., cloud masses, shorelines at different tidal stages, or eroded shorelines) were excluded from the image motion estimation. The tidal stage of the August 2011 image was higher than that of the September 2011 image hence excluding the main shoreline from the coregistration area allowed only within marsh features to be used for coregistration. Thus, the difference in shoreline edge did not impact the accuracy of coregistration.

### Landcover classification and oil mapping

Kokaly et al. [5] successfully mapped oil contamination along the shoreline after the BP-DWH using AVIRIS images acquired on July 31, September 14 and October 2 of 2010 over Barataria Bay. They used hydrocarbon absorption features centered at 1720 nm and 2300 nm as inputs to the USGS's Material Identification and Characterization Algorithm (MICA) to map oil presence [5].

To compare inter-annual change for potential recovery or further damage, we needed to do all our analyses on the co-registered images, hence we mapped oil contamination using the continuum removal technique [41] over the two oil absorption features (Figure 1) in four AVIRIS flightlines over Barataria Bay acquired in September 2010, and did not use the oil maps produced by Kokaly et al. [5]. However, we used Kokaly et al. [5] field measured oil penetration data to validate our oil maps. We then compared the oiled coastline between the two maps to quantify the agreement between them.

**Figure 3:**
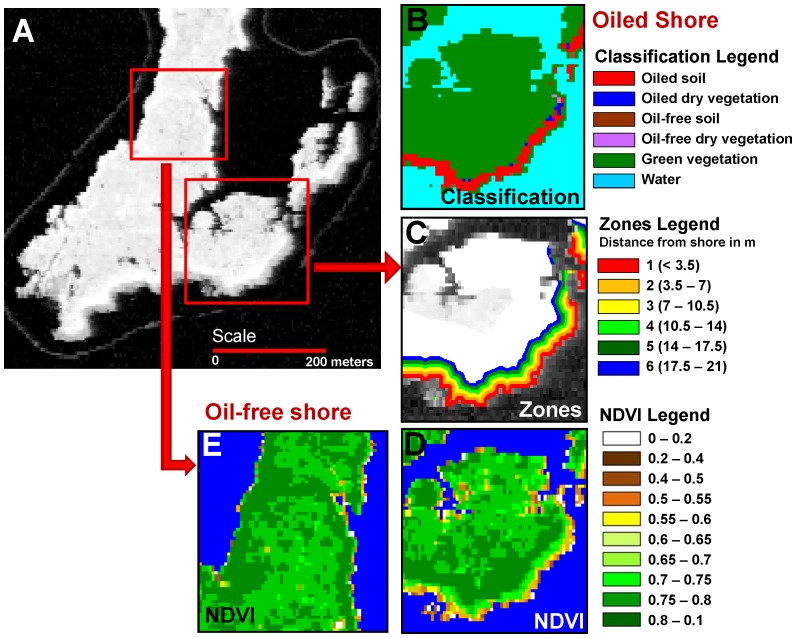
NDVI image, classified image and zones used to analyze oil impact. (a) Gray scale NDVI image of a subset of Barataria Bay, with a close up of an oiled section of the shoreline showing (b) the classified image, (c) the zones as we move away from the shoreline, and (d) the NDVI image showing the low-NDVI band of pixels right next to the oiled shoreline. For comparison, (e) shows the NDVI profile for an oil-free section of the shoreline.

The images were classified into six classes: water, soil, dry plant material or non-photosynthetic vegetation (NPV), photosynthetic vegetation, oiled soil and oiled NPV (Figure 3). We used a binary decision tree based on vegetation indexes, angle indexes (Table 1), and the depth of the oil absorption continuum removals to produce a classification map for the six classes following the methods in Khanna *et al*. [61]. In this case, the classification map was used to restrict vegetation stress analyses to the land class (i.e., every class except water). Clouds and cloud shadows were excluded from the analyses.

**Table 1:** Vegetation indexes used for stress detection due to oil contamination.

Inputs	Formula	Relevance	References
Normalized Difference Vegetation Index (NDVI)	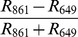	Index of green plant cover and LAI	[42]
Modified NDVI (mNDVI)	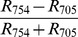	Sensitive to plant health	[47], [48]
Normalized Difference Infrared Index (NDII)	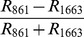	Sensitive to plant water content	[39], [40]
Angle at NIR (ANIR)	λ: 675, 1092, 1662 nm	Angle index sensitive to phase change in plants	[43]
Angle at Red (ARed)	λ: 550, 675, 1092 nm	Angle index sensitive to plant pigments	Adapted from [43]
Water Absorption at 980 nm (ADW1)	½* (*R*_783_ + *R*_1054_) – *R*_977_	Quantification of water absorption features	Adapted from [67]
Water Absorption at 1200 nm (ADW2)	½* (*R*_1054_+ *R*_1254_) – *R*_1187_	Quantification of water absorption features	Adapted from [67]

While we had sufficient field data to independently assess the accuracy of our oiled pixels, we did not have enough field data to train and validate our landcover map. To enrich our data set, and since we were mapping classes that are easily discernible by an observer, we collected training and validation data by visually interpreting photosynthetic vegetation, soil, water and NPV from the color infrared images. We randomly divided the September 2010 dataset into training and validation datasets and used the training dataset to train our classifier. Then we applied the same classifier to images from both dates. We assessed the accuracy of the oiled vs. oil-free pixels with data collected in the field using kappa statistics, overall classification accuracy and omission and commission errors [62], [63]. Accuracy for all classes was assessed using the visually interpreted validation data by calculating the same metrics.

### Comparing oiled vs. oil-free shoreline in September 2010

The boundary of the land class from the September 2010 image classification was used to produce a vector layer of the shoreline. This is because the September 2010 images were acquired at low-tide hence the shoreline generated by using this dataset represents the mean low tide water line. This shoreline was further simplified by deleting small polygons less than 500 m^2^ (small depressions within the islands filled with water) resulting in a smoother shoreline. We then calculated a “distance to shore” for every land pixel by computing the Euclidian distance and direction to shore in ArcGIS 10 (ESRI, Redlands, CA). We chose this approach instead of using a pre-existing shoreline vector layer because we wanted to compare the exact distances from the shoreline within our dataset, irrespective of how well the image was geo-registered.

#### Selection of pixels in oiled and oil-free shoreline zones

We selected pixels that were within 60 m of the oiled shoreline because, based on the USGS field data, we did not expect the oil to impact vegetation further inland. Further, to ensure that the pixels included were located in the section of the shoreline facing the ocean, we selected pixels with a direction perpendicular to the nearest shoreline between 0°–80° or 280°–360° (where 0° is North). This was done because tidal forces act differently on the ocean-facing southern shore compared to the east-west or northern shore. Oiled areas were predominantly located on the southern shoreline since the oil came in with the tide. Finally, only oil-free shorelines within 140 m of an oiled shoreline were used for comparison in order to maximize the similarity of environmental conditions between oiled and oil-free zones, except for the variable of interest, “oil”. Any bias would likely be in the direction of including oiled pixels in the oil-free area analysis which would result in minimizing differences between oiled and oil-free areas. Oil-free shoreline inland pixels were included following the same criteria as for oiled shorelines.

#### Vegetation index trajectories in oiled vs. oil-free shoreline zones

Plant physiological responses to stress often manifest as changes in chlorophyll and water content [64]. We selected seven indexes that track changes in chlorophyll content and/or leaf area of the plant (Normalized Difference Vegetation Index: NDVI, modified NDVI: mNDVI), change in plant condition (green, senescent, dead – Angle at NIR: ANIR, Angle at Red: ARed), and change in plant water content (Normalized Difference Infrared Index: NDII, Water Absorption at 980 nm: ADW1, Water Absorption at 1240 nm: ADW2) (Table 1). No single index is optimal for all conditions, so consistent responses across these diverse indexes would add robustness to our results. To determine the extent of oil-induced stress on interior marsh vegetation, we compared index values along oiled shorelines with those along oil-free shorelines for the September 2010 imagery. We used an analysis of variance to test whether there were significant differences between the indexes in oiled and oil-free shorelines, and then subdivided the data set by distance zones from the shoreline (Figure 3). Zones corresponded to the pixel size of the September 2010 images so that zone 1 was the first 3.5 m pixel next to the shore, zone 2 was the second pixel from the shore, etc. We tested for pair-wise differences in oiled versus oil-free shores in each zone (e.g., oil versus oil-free pixels at zone 1, oil versus oil-free pixels at zone 2, etc.) using a Tukey-HSD test [65]. This test showed the significance of all the possible pair-wise combinations of oiled versus oil-free zone comparisons that went into the overall ANOVA. Pixels were always compared within the same zone. We chose to compare corresponding zones because salt marsh species distributions follow the characteristics of flooding and elevation along shorelines. We observed a similar differentiating pattern in the remote sensing data, from lower index values in the intertidal region to a band of higher index values (robust dense vegetation) just beyond the intertidal and then slightly lower values again in the inner marsh. By grouping data by distance from the shorelines, we attempted to minimize changes in species types and density and their distribution patterns.

We tested the dependent variables, the index images, for autocorrelation between pixels using the Moran's I statistic. We found that the pixels were significantly autocorrelated until a distance of 21 m (Moran's I  = 0.48, p-value <<0.001). Despite this high correlation we decided not to subsample our data to include only independent pixels because of several reasons. We expected the first few pixels along the shoreline to be sensitive to oil and by choosing pixels far enough to be spatially independent, we could have missed detecting the impact of oil altogether. Moreover, the tests we used to determine if there was an impact are known to be robust to violations of the assumption of independence between samples [66]. Finally, a conservative approach can be undertaken to reduce the significance level from 0.05 to 0.01 and if significant differences are still found then despite the violation of the independence assumption, we are reassured that the differences are significant.

#### Post-classification comparison

Using the classification maps, wherein each pixel was assigned to a single class, we determined the number of photosynthetic vegetation pixels, and soil and NPV pixels within each zone along oiled and oil-free shorelines. If the impact of oil was discernible within a zone, we expected to see a smaller number of the pixels classified as photosynthetic vegetation along the oiled shoreline compared to pixels along the oil-free shoreline.

For both the analyses using index-trajectories and classification maps, we expected the vegetation along oiled and oil-free shorelines to be at the same phenological state barring the impact of oil because we were looking at a single date.

### Detecting recovery of stressed vegetation

#### Index values relative to distance from nearest oiled pixel

We used this analysis to determine potential continued decline or recovery from oil contamination a year later. Using our oil contamination map derived from the classification of the September 2010 imagery, we calculated the “distance to nearest oiled pixel” to determine the effect of proximity to oil contamination on plant stress. To test for significant differences in vegetation indexes we used an ANOVA with all the data (all zones combined) and a post-hoc Tukey-HSD test to determine how far from the oiled pixel we could see significant differences.

We used a linear regression for index values for the first four pixels away from the oiled pixels. At distances closer to an oiled pixel (up to 3 pixels away), we found a linear relationship between index values and distance from an oiled pixel. An asymptote was reached at distances farther away. Then we tested whether the slope of the linear regression was significantly different in August 2011 compared to September 2010 using a t-test and identified the direction of the change in slope. We expected a lower slope and weakened correlation by August 2011, which would indicate vegetation recovery, that is, the index values are no longer influenced by distance from oil. We should clarify that there was negligible oil in the August 2011 imagery. For both dates, “distance to nearest oiled pixel” relates to the original contamination in 2010. The only assumption we made was that there was no significant change in the location of oil contamination (like oil being carried further inland) after September 2010.

#### Post-classification change detection

We assessed whether there was continued vegetation decline or recovery a year later by looking at the percentage of pixels depleted of photosynthetic vegetation in September 2010 that were re-vegetated by August 2011. We also examined whether there was an effect of the position of the pixel relative to the shoreline in the extent of recovery, in other words, did recovery vary with distance from shore?

We were confident enough in our sub-pixel coregistration to do a pixel-to-pixel comparison of the classifications from both years. We quantified the number of pixels in each zone along oiled shorelines that were classified as soil or NPV in September 2010 but classified as photosynthetic vegetation in August 2011.

Although the 2010 imagery was collected in September and the 2011 imagery in August, thus during the same seasonal period, we *did* expect to see some differences in the phenological state of the wetland because of natural variation from year to year due to precipitation and temperature differences. However, because we were looking at a classified image rather than an index image, the data is categorized, not continuous. This minimizes small differences in plant vigor. As long as the plant is not in a completely different phenological state (e.g. green in 2010 to completely dry in 2011), the class of the pixel should not change. On the other hand, any recovery (new photosynthetic vegetation) in areas of denuded or dying vegetation will result in a class change. Thus recovery using this method does *not* indicate a return to “before oil” state but just that recovery has begun and there is fresh growth. This method is also not sensitive to declines in vegetation cover or health in areas adjacent to heavily oiled shorelines, that is, secondary oiling impacts.

## Results

### Classification Accuracy

Both 2010 and 2011 images were classified into six classes: soil, NPV, oiled soil, oiled NPV, photosynthetic vegetation and water (see Figure S1 for the full decision tree). The overall classification accuracy for the six classes in September 2010 was 98% and the Kappa coefficient was 0.97 (n = 427). For August 2011, the overall accuracy was 87.3% and Kappa was 0.8 (n = 3108). Table 2 shows confusion matrices for both dates using validation data distinct from training data used to build the classifier.

**Table 2:** Confusion matrices for September 2010 and August 2011 classifications.

	Ground Reference Data					
**September 2010 Confusion Matrix**						
**Classified as**	Oiled NPV	Green Veg.	Water	Oiled Soil	Total	User's Accuracy
Oiled NPV	**107**	**1**	**0**	**1**	**109**	**98.2**
Green Veg.	**3**	**139**	**0**	**2**	**144**	**96.5**
Water	**0**	**0**	**94**	**0**	**94**	**100.0**
Oiled Soil	**1**	**0**	**0**	**79**	**80**	**98.8**
Total	**111**	**140**	**94**	**82**	**427**	
Producer's Accuracy	**96.4**	**99.3**	**100.0**	**96.3**		**98.1**
**August 2011 Confusion Matrix**						
	Water	Green Veg.	NPV			
Water	**1185**	**1**	**0**		**1186**	**99.9**
Green Veg.	**0**	**1158**	**395**		**1553**	**74.6**
NPV	**0**	**0**	**369**		**369**	**100.0**
Total	**1185**	**1159**	**764**		**3108**	
Producer's Accuracy	**100.0**	**99.9**	**48.3**			**87.3**

Confusion matrices for classification of September 2010 and August 2011 imagery using user-interpreted data. In September 2010, there were no oil-free non-photosynthetic vegetation (NPV) and oil-free soil pixels and in August 2011, there were no oiled pixels in validation data.

Kokaly et al. [5] found that contamination due to the BP-DWH oil spill covered narrow zones along the shoreline. Our oil maps showed that average penetration into the marsh was 7.5 m while maximum penetration was up to 40 m. The accuracy of the classification of oiled pixels was assessed independently using data collected in the field by Kokaly et al. [5], [57]. When the classification map was collapsed down to two classes, “oil present” (oiled soil and oiled NPV) and “oil absent” (water, soil, NPV, and photosynthetic vegetation), the overall accuracy was 95% and the Kappa coefficient was 0.88 (n = 40, Table 3). Two of the field survey points had oil contamination but were classified as oil-free. Kokaly et al. [5] noted these areas to be lightly oiled with minimal penetration into the marsh. Comparison with the oil contamination map produced by Kokaly et al. [5] showed that 90.6% of the oiled shoreline mapped by Kokaly et al. [5] was classified as oiled in our map while 5.1% of the shoreline mapped as oiled in this study was classified as oil-free by Kokaly et al. [5]. Kokaly et al. [5] used multiple-date images (July, September and October, 2010) to map oiled pixels in all cover classes while our study concentrated on mapping oil in only the September, 2010 images.

**Table 3:** Confusion matrix for oiled vs. oil-free pixels in September 2010.

	Ground Reference Data			
Classified as	Oil-free	Oiled	Total	User's Accuracy
Oil-free	**10**	**2**	**12**	**83.3**
Oiled	**0**	**28**	**28**	**100.0**
Total	**10**	**30**	**40**	
Producer's Accuracy	**100.0**	**93.3**		**95.0**

Confusion matrices for classification of oiled pixels in September 2010 using field data collected by Kokaly et al. [5].

### Comparing oiled vs. oil-free shoreline for September 2010

#### Vegetation index trajectories

**Figure 4:**
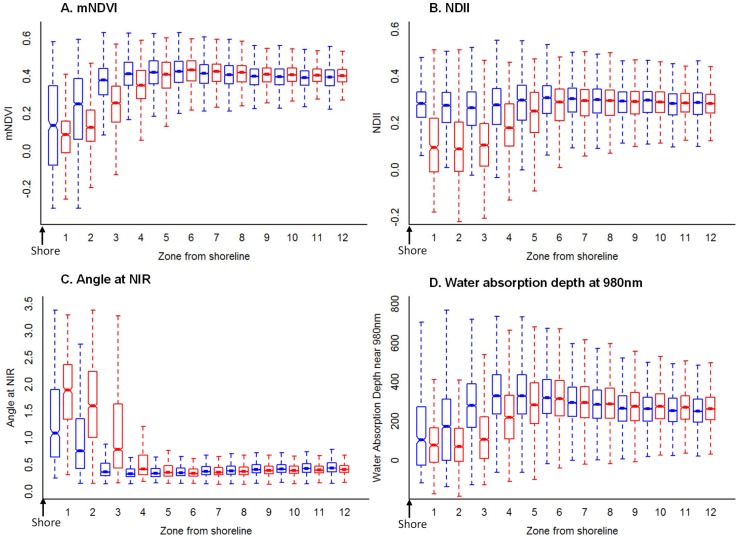
Index trajectories from shore to inland along oiled and oil-free shoreline. Box plots of index values for pixels vs. zone from shore (“1” being the first pixel from the shore and “12” being the twelfth) for oiled (red) and oil-free (blue) shoreline for the September 2010 dataset for (a) mNDVI, (b) NDII, (c) Angle at NIR, and (d) Absorption depth of water centered near 980nm. Distributions with non-overlapping notches are significantly different from each other. The notch represents the 95% confidence interval, the box extends from the first to the third quartile and the dashed lines extend to the data point which is 1.5 times the interquartile range.

In September 2010, index values were significantly different between oiled and oil-free shorelines, and index trajectories in the first four oiled shoreline zones (0 to 14 m) were significantly lower than in the corresponding oil-free shoreline zones (Figure 4, see also Tables S1 and S2). The ANIR index shows the opposite pattern to the other indexes, because lower values for this index indicate photosynthetic vegetation (the angle at NIR gets narrower in healthy vegetation).

#### Post-classification comparison

**Figure 5:**
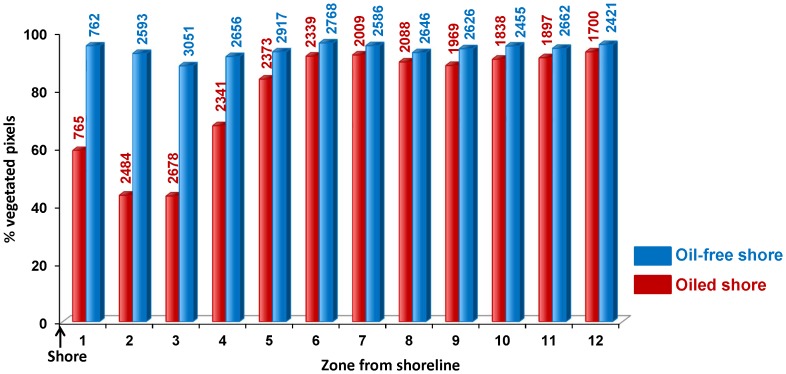
Photosynthetic vegetation cover from shore to inland along oiled and oil-free shoreline. Percentage of photosynthetic vegetation pixels in oiled vs. oil-free shoreline zones in September 2010 (“1” being the first pixel from the shore and “12” being the twelfth). Total number of pixels for each zone for oiled and oil-free shoreline is shown above the bars.

The impact extended four pixels inland (0–14 m from shore). The percentage of pixels classified as photosynthetic vegetation was lower in the first four zones (<60%) in oiled shorelines relative to the corresponding zones in oil-free shorelines (>90%), consistent with observations on the index value trajectories (Figure 5). By the fifth pixel from the shore, the difference in percent of photosynthetic vegetation along oiled vs. oil-free shoreline was much narrower (83.8% vs. 93.2% or 9.4% difference). Beyond the first five pixels, the percentage of photosynthetic vegetation pixels along oiled and oil-free shorelines was similar and above 90%. Pixels in the second and third zone away from the shoreline were the most affected by oil (had the lowest percentage of photosynthetic vegetation pixels) followed by pixels adjacent to the shoreline.

### Detecting recovery of stressed vegetation

#### Distance to nearest oiled pixel

**Figure 6:**
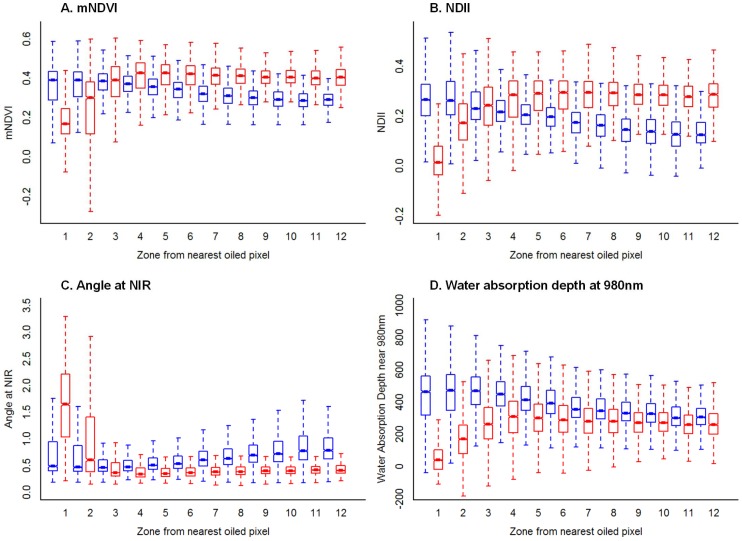
Index trajectories w.r.t. distance to the nearest oiled pixel in September 2010 and August 2011. Box plots of index values for pixels vs. distance from nearest oiled pixel (“1” being an oiled pixel and “12” being 12 pixels away from the nearest oiled pixel) in September 2010 (red) and August 2011 (blue) for (a) mNDVI, (b) NDII, (c) Angle at NIR, and (d) Absorption depth of water centered near 980 nm. Distributions with non-overlapping notches are significantly different from each other. The notch represents the 95% confidence interval, the box extends from the first to the third quartile and the dashed lines extend to the data point which is 1.5 times the interquartile range.

In September 2010, index values were lowest in pixels closest to oiled pixels and increased as distance to the nearest oiled pixel increased (Figure 6, see also Tables S3 and S4). The impact of oil was observed up to 7 m from the nearest oiled pixel (up to two pixels away). All index values significantly increased as distance to the nearest oiled pixel increased in the first three pixels away from an oiled pixel (Table 4), with the exception of ANIR (which showed the opposite trend in values, thereby indicating the same pattern in plant stress). However, the amount of variance explained by this variable was not very high (R^2^ between 0.11 to 0.42, Table 4).

**Table 4:** Regression analysis of September 2010 and August 2011 indexes.

	September 2010			August 2011				
Index	Slope coefficient	Coefficient of determination (R^2^)	F value*	Slope coefficient	Coefficient of determination (R^2^)	F value*		Sep 2010 vs. Aug 2011: T value**
NDVI	**0.06**	**0.11**	**1780**	**0.02**	**0.01**		**122**	17.9
mNDVI	**0.07**	**0.20**	**3692**	**0.01**	**0.00**		**53**	43.1
NDII	**0.08**	**0.42**	**10360**	**−0.01**	**0.02**		**324**	87.5
ANIR	**−0.37**	**0.29**	**5733**	**−0.08**	**0.01**		**145**	−37.1
ARed	**0.49**	**0.34**	**7521**	**−0.08**	**0.02**		**286**	73.0
ADW1	**80.5**	**0.27**	**5386**	**7.77**	**0.00**		**2949**	40.3
ADW2	**119.8**	**0.28**	**5597**	**26.58**	**0.01**		**151**	34.6

* All regressions were significant (p-value <0.001)

** All 2010 vs. 2011 slopes were significantly different (p-value < 0.001)

Regression analysis of September 2010 and August 2011 indexes data with respect to “distance from the nearest oiled pixel” (n = 14,372).

By August 2011, less than 1% of the variance was explained by the linear relationship between proximity to oil and index values (R^2^<0.05 for all indexes, Table 4). The slopes were also significantly lower in August 2011 than those in September 2010 for all indexes (p-value <0.001, Table 4). Figure 6 illustrates the steep slope in the first three zones in September 2010 relative to August 2011.

#### Post-classification change detection

**Figure 7:**
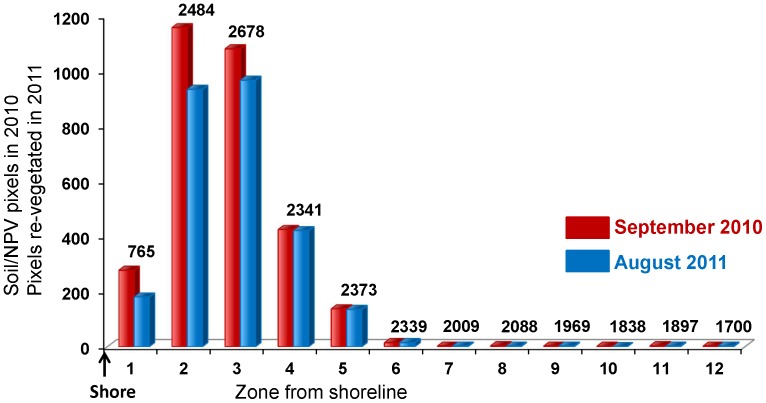
Recovery of photosynthetic vegetation from September 2010 to August 2011. The number of oiled soil & NPV pixels in September 2010 that recovered (regrowth of vegetation) a year later in August 2011 in pixels next to the shore (“1” being the first pixel from the shore and “12” being the twelfth). Total number of pixels for each zone is shown above the bars.

When comparing September 2010 and August 2011 classifications, we found that recovery or re-vegetation of pixels classified as dead or dying vegetation in 2010 was poorest closer to the shoreline, with a lower percentage of re-vegetated pixels, improving with increased distance from the shore. By the fourth pixel, almost all pixels that were oiled and denuded of photosynthetic vegetation in September 2010 had recovered and were classified as photosynthetic vegetation in August 2011 (Figure 7).

## Discussion

### Impact of oil contamination

The indexes we used in this study are indicators of important biophysiological properties of plants such as chlorophyll concentration, leaf area, plant water, and plant phenology [34], [35], [36], [37], [38]. Loss of pigment, water, and leaves are all symptoms of plant stress. We found that vegetation was more stressed along oiled shoreline in September 2010 compared to oil-free shoreline. This was true within every zone for at least the first four zones adjacent to the shoreline. Thus, the oil-impacted zone along the shoreline was 14 m. Kokaly et al. [5] noted that mean oil penetration in the region was 11 m. Our results show that the actual impact of oil on vegetation extends beyond the region where oil penetration is visible in the image.

We also observed in the field that when oil is carried inland through tidal action, it can contaminate the soil under the canopy while the plant canopy surface has no oil. Plant mortality is much higher when leaves are coated by oil than when the oil is present below the canopy only in the soil layer [1], [7], [12]. Therefore, we expected the impact of oil to decrease as we moved inland from the shoreline. This was confirmed by the general trajectory of index values with respect to the shoreline. Index values were lowest (indicating high plant stress) in the zone next to the shoreline but increased as we moved away from the shoreline, stabilizing at about 14 m from the shore. While significant differences were found only in the first four zones (five for water absorption bands), this does not mean that there was no impact beyond this zone. Some places where oil had penetrated much further inland likely showed impact far beyond 14 m but since the analysis pooled data from the entire study area, localized areas that may have been affected further inland did not result in a significant difference.

When we examined index behavior with respect to distance to the nearest oiled pixel, the response can be divided into two segments: the first 2–3 pixels from the nearest oiled pixel have an approximately linear response (index values increase with increasing distance) while pixels farther than that seem independent of distance from shore (see Figure 6). This is observed in September 2010 along the oiled shorelines by the significantly positive slopes of mNDVI, NDII, and ADW1 (negative slope for ANIR) as distance from oil increases. Plant stress extended, on average, 7 m inland from the closest oiled pixels. This gradient in impact was also likely due to the same reasons described above. The sub-canopy oil did not seem to have a measurable impact on the photosynthetic ability of the plants beyond this distance.

Comparison of classified pixels along oiled and oil-free shorelines showed that the lowest percentage of pixels containing photosynthetic vegetation occurred in the first three pixels away from the shoreline. This pattern is likely due to the fact that plants near the shoreline had their leaves and stems completely flooded by oil, hence plant mortality was very high [57]. Zonal bands, 1–2 pixels away from the shoreline (3.5 to 10.5 m) had a lower percentage of photosynthetic vegetation pixels compared to the pixels adjacent to the shore. We suspect this was because many of the oil absorbing floating booms that were used to prevent oil from reaching the shore may have washed onto the marshes and settled at the high tide line, within the 1–2 pixels from the shoreline, corresponding to the zone where plant mortality was highest (personal observation in October, 2010). The booms that were transported into the marsh were generally saturated with oil and thereby increased the intensity and residence time of oil contamination near the high tide line which can lead to higher impact on the plant community [2], [11], [12]. Moreover, frequency of tidal action decreases as we move away from the shore. This also leads to longer residence time for the oil. Another possible reason for greater impact in the pixels 3.5 to 10.5 m from shore is that water levels were higher when the oil came ashore and as the water receded, it deposited more oil inland than in the zone adjacent to the shore.

### Detecting recovery of stressed vegetation

Our results indicate that tidal marsh communities were beginning to recover in August 2011 because their spectral index values were no longer influenced by proximity to oil contamination. *S. alterniflora*, the dominant salt marsh species along the shoreline in Barataria Bay, is relatively robust to oil contamination in terms of above-ground biomass, stem density and nutrient uptake [1], [7], [12]. *S. patens* is relatively more sensitive to oil but is found further inland in the brackish marsh. Recovery in above-ground biomass in both species is quick. DeLaune and Wright [12] note that *S. alterniflora* recovers within a year even when the leaves have been oiled and the canopy has died.

A comparison of the classifications of oiled and oil-free shorelines in September 2010 imagery indicated that the first 10.5 m along oiled shorelines had a high percentage of oiled bare soil and oiled NPV pixels (as high as 50%) potentially indicating areas that were denuded of vegetation as a result of oil-induced plant stress. By 24 m inland from shore, the number of oiled bare soil and NPV pixels was negligible (<1%). In August 2011, recovery was the poorest in pixels adjacent to the shore (65% of oiled soil and NPV pixels contained photosynthetic vegetation in the following year), followed by the next two zones, less than 7 m from shore (more than 80% of the oiled soil and NPV pixels in the second zone from the shore contained photosynthetic vegetation in the following year) and by the fourth zone (14 m from shore), re-vegetation was almost 100% supporting the conclusion that much of the inland marsh had regenerated. Hester and Mendelssohn [7] observed in a five year study of a Louisiana brackish marsh recovering from oil contamination that failure to re-vegetate was more often due to lack of elevation (resulting in waterlogging stress) than directly due to the oil. Elevation is lowest in pixels adjacent to the shoreline, and additionally, it is subject to erosion as a result of denuded vegetation. This probably led to poor recovery in pixels next to the shoreline.

The difference in the range of index values beyond the impact zone between September 2010 and August 2011 was likely due to 2011 being a much drier year than 2010, and not oil impacts. Precipitation in 2011 (754 mm) was only one-third of 2010 levels (2233 mm; National Climate Data Center) resulting in lower overall index values and a drier interior marsh relative to the near-shore marsh. This interpretation is further supported by the absence of large storms that would have been powerful enough to bring tidal water farther into the marsh.

This study clearly showed that the impact of oil characterized using vegetation indexes extended no further than a couple of pixels from a contaminated pixel. While Mishra et al. [4] found oil impacts to be far more widespread (>400 km^2^), the broad spatial and spectral resolution of the Landsat imagery they used prevented the direct detection of oil. Instead, they compared the 2009 growing season to the 2010 growing season to determine extent of oil impact. The large areas of vegetation change mapped with 30 m pixels could be due to other reasons such as precipitation differences, natural variation in vegetation phenology from year to year, etc. The impacted areas included salt marshes (e.g. Barataria Bay), brackish and freshwater marshes (e.g. Bird's Foot) and mangroves (e.g. Chandeleur Islands). Some of these areas are far more fragmented by oil and gas infrastructure than others. Species composition also changes considerably among different vegetation types. Thus they may show considerable variation in oil impact.

There was a loss of 15% to 30% vegetated pixels in 2011 that changed to water from photosynthetic vegetation, residue and soil classes. According to local tide tables for these two dates and the time that the images were flown, the tidal stage in August 2011 was about 0.12 m higher than in September 2010 (tides.rodnreel.com). In September 2010, time of data collection coincided with the low tide (0.18 m), while in August 2011, data were collected only slightly below high tide levels (0.30 m). Hence, the increase in water pixels in August 2011 was in part due to tidal differences between the two dates.

## Conclusions

The full impact of the BP-DWH oil spill on the marshland, mangroves and wildlife of the region is still under study. We used AVIRIS imagery collected over Barataria Bay in September 2010 and August 2011 to evaluate whether vegetation health in these marshes continued to decline or experienced recovery in the following year. We successfully assessed marsh impacts of the oil spill finding that the marsh showed negative impacts due to oil exposure but that some recovery occurred within one year. The impact was contingent on distance to oil contamination. We also found that sub-canopy oil did not seem to have a discernable effect on marsh communities beyond a couple of pixels. While this is encouraging news for marsh health, we don’t know whether different plant communities respond differently. Further we cannot address how this immediate impact cascades through the ecosystem.

This analysis is based on two AVIRIS snapshots in time, September 2010 and August 2011. Recovery in the marsh is likely still ongoing. Moreover, the strong storm surge brought in by hurricane Isaac in 2012 might have remobilized oil and brought it further inland. More research is needed to determine long-term impacts of the oil spill. However, this study illustrates that high spatial resolution imaging spectroscopy serves as a powerful tool to establish the link between oil contamination and plant stress and to document plant die-off and recovery in the marsh after the disaster.

## Supporting Information

Figure S1 **Decision tree for classification of AVIRIS imagery.** Decision tree showing multiple variables used at each node to separate one target class from the rest of the classes. Oiled pixels were classified using continuum removal (CR) over two oil absorptions at 1720 nm and 2300 nm in only soil and non-photosynthetic vegetation (NPV) pixels. R_x_ indicates reflectance in the “x” region of the electromagnetic spectrum.
(TIFF)

Table S1 **Mean index values w.r.t distance to shore in September 2010.** Mean values for seven indexes vs. distance to shore for oiled and oil-free shorelines for the September 2010 dataset (index acronyms are listed in table 1).
(DOCX)

Table S2 **Index analysis for oiled vs. oil-free zones w.r.t distance to shore in September 2010.** Analysis of variance between oil versus oil-free shorelines in September 2010 and per zone comparisons (index acronyms are listed in table 1). Degrees of freedom  = 185,887.
(DOCX)

Table S3 **Mean index values w.r.t distance to nearest oiled pixel.** Mean values for seven indexes vs. distance to nearest oiled pixel for oiled shoreline in September 2010 and a year later in August 2011.
(DOCX)

Table S4 **Index analysis for Sept. 2010 vs. Aug. 2011 oiled zones w.r.t distance from nearest oiled pixel.** Analysis of variance between pixels at different distances from an oiled pixel in September 2010, and Tukey HSD tests for pair-wise comparisons (index acronyms are listed in Table 1). Degrees of freedom  = 114,371.
(DOCX)
