# Supplementary figures and images for: Detection of Salt Marsh Vegetation Stress and Recovery after the Deepwater Horizon Oil Spill in Barataria Bay, Gulf of Mexico Using AVIRIS Data
Source: PLoS One. 2013 Nov 5;8(11):e78989. doi: 10.1371/journal.pone.0078989 (PMC3818498; doi:10.1371/journal.pone.0078989)

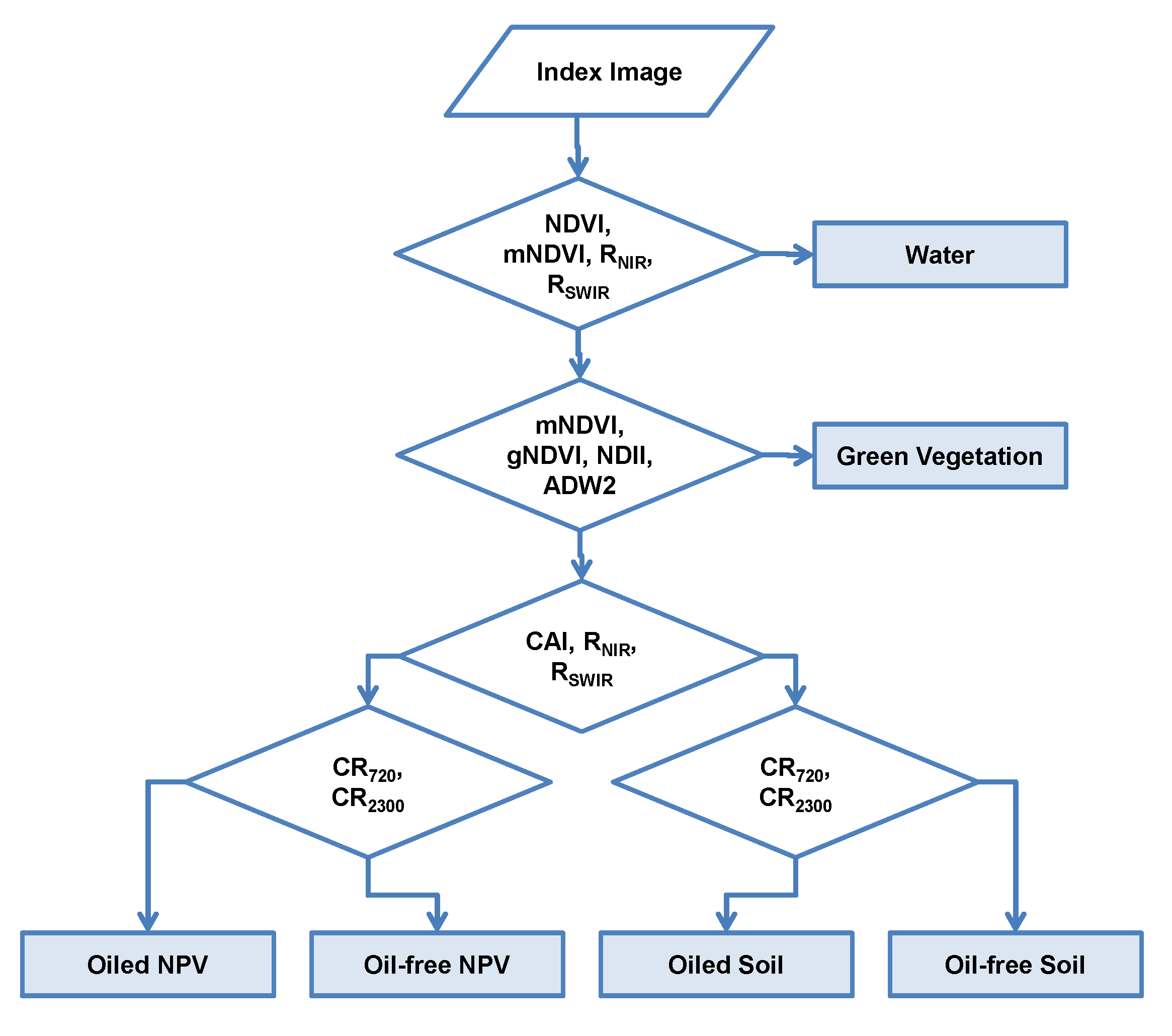

Supplement: Figure S1 — Decision tree for classification of AVIRIS imagery. Decision tree showing multiple variables used at each node to separate one target class from the rest of the classes. Oiled pixels were classified using continuum removal (CR) over two oil absorptions at 1720 nm and 2300 nm in only soil and non-photosynthetic vegetation (NPV) pixels. Rx indicates reflectance in the “x” region of the electromagnetic spectrum. (TIFF) [file pone.0078989.s001.tiff]
