# Supplementary material for: Detection of Salt Marsh Vegetation Stress and Recovery after the Deepwater Horizon Oil Spill in Barataria Bay, Gulf of Mexico Using AVIRIS Data
Source: PLoS One. 2013 Nov 5;8(11):e78989. doi: 10.1371/journal.pone.0078989 (PMC3818498; doi:10.1371/journal.pone.0078989)
Supplement: Table S1 — Mean index values w.r.t distance to shore in September 2010. Mean values for seven indexes vs. distance to shore for oiled and oil-free shorelines for the September 2010 dataset (index acronyms are listed in table 1). (DOCX) [file pone.0078989.s002.docx]

**Table S1: Mean index values w.r.t distance to shore in September 2010**

**Legend:** Mean values for seven indexes vs. distance to shore for oiled and oil-free shorelines for the September 2010 dataset (index acronyms are listed in table 1).

| **Zone from Shoreline** | | **N** | **NDVI** | **mNDVI** | **NDII** | **ANIR** | **ARed** | **ADW1** | **ADW2** |
| --- | --- | --- | --- | --- | --- | --- | --- | --- | --- |
| **OILED SHORELINE, September 2010** | | | | | | | | | |
| **1** | **765** | | **0.368** | **0.079** | **0.105** | **1.841** | **4.021** | **86** | **153** |
| **2** | **2484** | | **0.434** | **0.127** | **0.100** | **1.613** | **3.916** | **89** | **181** |
| **3** | **2678** | | **0.583** | **0.253** | **0.115** | **1.060** | **4.075** | **133** | **306** |
| **4** | **2341** | | **0.658** | **0.347** | **0.185** | **0.643** | **4.500** | **228** | **463** |
| **5** | **2373** | | **0.693** | **0.393** | **0.241** | **0.470** | **4.916** | **293** | **544** |
| **6** | **2339** | | **0.717** | **0.418** | **0.273** | **0.405** | **5.155** | **321** | **577** |
| **7** | **2009** | | **0.715** | **0.413** | **0.280** | **0.415** | **5.179** | **311** | **558** |
| **8** | **2088** | | **0.714** | **0.408** | **0.280** | **0.420** | **5.188** | **302** | **540** |
| **9** | **1969** | | **0.708** | **0.400** | **0.274** | **0.443** | **5.140** | **286** | **515** |
| **10** | **1838** | | **0.708** | **0.398** | **0.277** | **0.446** | **5.176** | **284** | **510** |
| **11** | **1897** | | **0.710** | **0.400** | **0.280** | **0.440** | **5.213** | **281** | **505** |
| **12** | **1700** | | **0.709** | **0.400** | **0.282** | **0.446** | **5.249** | **283** | **505** |
| **OIL-FREE SHORELINE, September 2010** | | | | | | | | | |
| **1** | **762** | | **0.409** | **0.134** | **0.269** | **1.303** | **4.636** | **133** | **243** |
| **2** | **2593** | | **0.495** | **0.209** | **0.259** | **1.001** | **4.818** | **171** | **322** |
| **3** | **3051** | | **0.658** | **0.356** | **0.255** | **0.506** | **5.072** | **286** | **513** |
| **4** | **2656** | | **0.699** | **0.400** | **0.269** | **0.422** | **5.160** | **345** | **593** |
| **5** | **2917** | | **0.709** | **0.411** | **0.284** | **0.408** | **5.243** | **343** | **586** |
| **6** | **2768** | | **0.717** | **0.416** | **0.297** | **0.407** | **5.304** | **334** | **570** |
| **7** | **2586** | | **0.714** | **0.411** | **0.297** | **0.423** | **5.307** | **321** | **551** |
| **8** | **2646** | | **0.707** | **0.400** | **0.288** | **0.447** | **5.266** | **303** | **523** |
| **9** | **2626** | | **0.702** | **0.395** | **0.288** | **0.459** | **5.265** | **290** | **501** |
| **10** | **2455** | | **0.704** | **0.395** | **0.287** | **0.452** | **5.265** | **284** | **494** |
| **11** | **2662** | | **0.696** | **0.387** | **0.283** | **0.475** | **5.238** | **269** | **472** |
| **12** | **2421** | | **0.696** | **0.387** | **0.285** | **0.484** | **5.262** | **267** | **466** |
