# Supplementary material for: Detection of Salt Marsh Vegetation Stress and Recovery after the Deepwater Horizon Oil Spill in Barataria Bay, Gulf of Mexico Using AVIRIS Data
Source: PLoS One. 2013 Nov 5;8(11):e78989. doi: 10.1371/journal.pone.0078989 (PMC3818498; doi:10.1371/journal.pone.0078989)
Supplement: Table S2 — Index analysis for oiled vs. oil-free zones w.r.t distance to shore in September 2010. Analysis of variance between oil versus oil-free shorelines in September 2010 and per zone comparisons (index acronyms are listed in table 1). Degrees of freedom = 185,887. (DOCX) [file pone.0078989.s003.docx]

**Table S2: Index analysis for oiled vs. oil-free zones w.r.t distance to shore in September 2010**

**Legend:** Analysis of variance between oil versus oil-free shorelines in September 2010 and per zone comparisons (index acronyms are listed in table 1). Degrees of freedom = 185,887.

|  |  |  | Tukey HSD pair-wise comparisons (p-value) | | | | |
| --- | --- | --- | --- | --- | --- | --- | --- |
|  | F-test | p-value | Zone 1 (0-3.5m) | Zone 2 (3.5-7m) | Zone 3 (7-10.5m) | Zone 4 (10.5-14m) | Zone 5-12 (>14m) |
| NDVI | 312.1 | <<0.001 | <<0.001 | <<0.001 | <<0.001 | 0.001 | >0.05 |
| mNDVI | 585.7 | <<0.001 | <<0.001 | <<0.001 | <<0.001 | 0.001 | >0.05 |
| NDII | 4112.0 | <<0.001 | <<0.001 | <<0.001 | <<0.001 | 0.001 | >0.05 |
| ANIR | 989.9 | <<0.001 | <<0.001 | <<0.001 | <<0.001 | 0.001 | >0.05 |
| Ared | 1994.0 | <<0.001 | <<0.001 | <<0.001 | <<0.001 | 0.001 | >0.05 |
| ADW1 | 572.2 | <<0.001 | <<0.001 | <<0.001 | <<0.001 | 0.001 | >0.05 |
| ADW2 | 122.5 | <<0.001 | <<0.001 | <<0.001 | <<0.001 | 0.001 | >0.05 |
