# Supplementary material for: Detection of Salt Marsh Vegetation Stress and Recovery after the Deepwater Horizon Oil Spill in Barataria Bay, Gulf of Mexico Using AVIRIS Data
Source: PLoS One. 2013 Nov 5;8(11):e78989. doi: 10.1371/journal.pone.0078989 (PMC3818498; doi:10.1371/journal.pone.0078989)
Supplement: Table S3 — Mean index values w.r.t distance to nearest oiled pixel. Mean values for seven indexes vs. distance to nearest oiled pixel for oiled shoreline in September 2010 and a year later in August 2011. (DOCX) [file pone.0078989.s004.docx]

**Table S3:** **Mean index values w.r.t distance to nearest oiled pixel**

**Legend:** Mean values for seven indexes vs. distance to nearest oiled pixel for oiled shoreline in September 2010 and a year later in August 2011.

| **Zone from oiled pixel** | **N** | **NDVI** | **mNDVI** | **NDII** | **ANIR** | **ARed** | **ADW1** | **ADW2** |
| --- | --- | --- | --- | --- | --- | --- | --- | --- |
| **September 2010** | | | | | | | | |
| **1** | **3457** | **0.518** | **0.174** | **0.022** | **1.635** | **3.622** | **53** | **170** |
| **2** | **3675** | **0.529** | **0.238** | **0.173** | **0.985** | **4.294** | **174** | **343** |
| **3** | **3784** | **0.634** | **0.340** | **0.234** | **0.611** | **4.819** | **261** | **485** |
| **4** | **3457** | **0.672** | **0.375** | **0.263** | **0.518** | **5.079** | **292** | **521** |
| **5** | **3674** | **0.680** | **0.380** | **0.271** | **0.503** | **5.129** | **288** | **512** |
| **6** | **4087** | **0.684** | **0.382** | **0.277** | **0.496** | **5.154** | **287** | **511** |
| **7** | **3956** | **0.688** | **0.385** | **0.278** | **0.490** | **5.163** | **289** | **506** |
| **8** | **4298** | **0.688** | **0.385** | **0.281** | **0.492** | **5.190** | **287** | **502** |
| **9** | **4377** | **0.688** | **0.384** | **0.281** | **0.498** | **5.180** | **283** | **495** |
| **10** | **4376** | **0.693** | **0.388** | **0.283** | **0.487** | **5.217** | **285** | **498** |
| **11** | **4569** | **0.693** | **0.387** | **0.280** | **0.486** | **5.225** | **278** | **486** |
| **12** | **4820** | **0.697** | **0.390** | **0.282** | **0.474** | **5.258** | **280** | **488** |
| **August 2011** | | | | | | | | |
| **1** | **3457** | **0.317** | **0.268** | **0.943** | **5.178** | **5.178** | **424** | **646** |
| **2** | **3675** | **0.480** | **0.316** | **0.269** | **0.951** | **5.061** | **436** | **667** |
| **3** | **3784** | **0.530** | **0.338** | **0.248** | **0.794** | **4.979** | **453** | **719** |
| **4** | **3457** | **0.550** | **0.340** | **0.230** | **0.736** | **4.906** | **444** | **716** |
| **5** | **3674** | **0.548** | **0.332** | **0.214** | **0.741** | **4.788** | **423** | **694** |
| **6** | **4087** | **0.540** | **0.320** | **0.202** | **0.767** | **4.699** | **401** | **669** |
| **7** | **3956** | **0.530** | **0.310** | **0.191** | **0.811** | **4.617** | **386** | **648** |
| **8** | **4298** | **0.523** | **0.302** | **0.183** | **0.834** | **4.549** | **374** | **628** |
| **9** | **4377** | **0.518** | **0.299** | **0.179** | **0.852** | **4.508** | **370** | **623** |
| **10** | **4376** | **0.519** | **0.296** | **0.168** | **0.859** | **4.445** | **363** | **614** |
| **11** | **4569** | **0.520** | **0.294** | **0.161** | **0.863** | **4.420** | **356** | **605** |
| **12** | **4820** | **0.514** | **0.286** | **0.153** | **0.895** | **4.357** | **346** | **590** |
