# Supplementary material for: Detection of Salt Marsh Vegetation Stress and Recovery after the Deepwater Horizon Oil Spill in Barataria Bay, Gulf of Mexico Using AVIRIS Data
Source: PLoS One. 2013 Nov 5;8(11):e78989. doi: 10.1371/journal.pone.0078989 (PMC3818498; doi:10.1371/journal.pone.0078989)
Supplement: Table S4 — Index analysis for Sept. 2010 vs. Aug. 2011 oiled zones w.r.t distance from nearest oiled pixel. Analysis of variance between pixels at different distances from an oiled pixel in September 2010, and Tukey HSD tests for pair-wise comparisons (index acronyms are listed in Table 1). Degrees of freedom = 114,371. (DOCX) [file pone.0078989.s005.docx]

**Table S4:** **Index analysis for Sept. 2010 vs. Aug. 2011 oiled zones w.r.t distance from nearest oiled pixel**

**Legend:** Analysis of variance between pixels at different distances from an oiled pixel in September 2010, and Tukey HSD tests for pair-wise comparisons (index acronyms are listed in Table 1). Degrees of freedom = 114,371.

|  |  |  | Tukey HSD pair-wise comparisons (p-value) | | | | |
| --- | --- | --- | --- | --- | --- | --- | --- |
|  | F-test | p-value | Zone 1 (0-3.5m) | Zone 2 (3.5-7m) | Zone 3 (7-10.5m) | | Zone 4 (10.5-14m) |
| NDVI | 1780.0 | <<0.001 | <<0.001 | <<0.001 | 0.001 | >0.05 | |
| mNDVI | 3692.0 | <<0.001 | <<0.001 | <<0.001 | 0.001 | >0.05 | |
| NDII | 10360.0 | <<0.001 | <<0.001 | <<0.001 | 0.001 | >0.05 | |
| ANIR | 5733.0 | <<0.001 | <<0.001 | <<0.001 | 0.001 | >0.05 | |
| Ared | 7521.0 | <<0.001 | <<0.001 | <<0.001 | 0.001 | >0.05 | |
| ADW1 | 5386.0 | <<0.001 | <<0.001 | <<0.001 | 0.001 | >0.05 | |
| ADW2 | 5597.0 | <<0.001 | <<0.001 | <<0.001 | 0.001 | >0.05 | |
